# Responses of neurons in the rat’s inferior colliculus to a sound are affected by another sound in a space-dependent manner

**DOI:** 10.1038/s41598-019-50297-8

**Published:** 2019-09-26

**Authors:** Mathiang G. Chot, Sarah Tran, Huiming Zhang

**Affiliations:** Department of Biomedical Sciences, University of Windsor, Windsor, Ontario N9B 3P4 Canada

**Keywords:** Midbrain, Inhibition-excitation balance, Sensory processing, Neurophysiology

## Abstract

The perception of a sound can be influenced by another sound in a space-dependent manner. An understanding of this perceptual phenomenon depends on knowledge about how the spatial relationship between two sounds affects neural responses to the sounds. We used the rat as a model system and equal-probability two-tone sequences as stimuli to evaluate how spatial separation between two asynchronously recurring sounds affected responses to the sounds in midbrain auditory neurons. We found that responses elicited by two tone bursts when they were colocalized at the ear contralateral to the neuron were different from the responses elicited by the same sounds when they were separated with one at the contralateral ear while the other at another location. For neurons with transient sound-driven firing and not responsive to stimulation presented at the ipsilateral ear, the response to a sound with a fixed location at the contralateral ear was enhanced when the second sound was separated. These neurons were likely important for detecting a sound in the presence of a spatially separated competing sound. Our results suggest that mechanisms underlying effects of spatial separation on neural responses to sounds may include adaptation and long-lasting binaural excitatory/inhibitory interaction.

## Introduction

A natural acoustic environment typically contains multiple sounds with their qualities, locations, and timing being different from each other. The perception produced by a sound in such an environment can be affected by another sound in a space-dependent manner. For instance, a speech sound can be masked by an interfering sound and the effect of masking can be reduced by spatial separation between the sounds^1–5^. Knowledge about how the spatial relationship between two sounds shapes neural responses to the sounds is needed for understanding hearing in a natural environment that contains multiple sounds^6^.

The midbrain auditory structure, the inferior colliculus (IC), is important for the processing of spatial acoustic cues^7,8^. This structure receives convergent inputs from both the left and right auditory pathways^9–11^. Interaction between these inputs (i.e., binaural interaction) enables neurons in the IC to compare acoustic cues obtained at the two ears^12–14^. In the rat, a rodent species with high-frequency hearing, major inputs to the IC driven by the contralateral ear are excitatory while those driven by the ipsilateral ear are inhibitory^15–19^. Thus, most of the neurons in the IC generate stronger firing in response to a dichotic stimulus when the difference between the two ears in sound-pressure level (i.e., interaural-level difference) favors the contralateral than the ipsilateral ear^20–23^. Although yet to be tested experimentally, it is possible that these neurons can generate stronger firing in response to a sound when it is from the contralateral than the ipsilateral acoustic field. This possibility is supported by results from other rodent species that have similar hearing sensitivities and auditory neural circuitry^24,25^.

The response of an IC neuron to a target sound and the spatial receptive field of an IC neuron can be affected by a simultaneously presented interfering sound in an acoustic environment^26–29^. Binaural interaction likely plays a key role in generating such space-dependent effects^27,28^.

Binaural interaction in the IC can occur even if the two ears are not stimulated simultaneously. In the rat’s IC, a leading ipsilateral tone burst can enhance or suppress the response of a neuron to a trailing contralateral tone burst presented tens or even hundreds of milliseconds later^30^. In agreement with this finding, studies using free-field and virtual-acoustic-space stimulation revealed that a leading sound can affect the response of an IC neuron to a spatially separated trailing sound as well as the spatial tuning of a neuron evaluated by the trailing sound^31–33^. Although adaptation may be involved in shaping the response to a trailing sound, the complexity of the effect of a leading sound suggests that other mechanisms such as excitatory-inhibitory interaction may also be involved.

Sounds in a natural environment can occur repeatedly at their respective timing. Ongoing interaction can exist among such recurring sounds in the generation of responses in auditory neurons. It is conceivable that the interaction between two sounds presented as recurring stimuli is different from that between the same sounds presented as a pair of leading-trailing stimuli.

In the present study, we used an equal-probability two-tone sequence to study the interaction between two independently recurring sounds in the generation of neural responses in the rat’s IC. We compared responses elicited by such a sequence when the two sounds were colocalized at the ear contralateral to a neuron in the IC and when they were spatially separated. We found that the response of a neuron to one sound in the existence of the other sound was enhanced by spatial separation between the sounds. The enhancement was particularly large in a group of neurons with transient firing and irresponsive to stimulation presented at the ipsilateral ear.

## Results

### Basic characteristics of responses

Eighty-eight neurons were recorded from the IC of 34 rats. In response to tone bursts presented at c90°, these neurons displayed characteristic frequencies (CFs) ranging from 0.8 to 49.0 kHz with the median at 6.0 kHz (Supplementary Fig. S1). They had transient (n = 53), sustained (n = 34), or offset (n = 1) patterns of firing in response to a CF tone burst presented at c90° (Supplementary Fig. S2). The distribution of CFs was similar between transient- and sustained-firing neurons (Supplementary Fig. S1, Mann-Whitney test, U = 861.50, p = 0.731).

**Figure 1:**
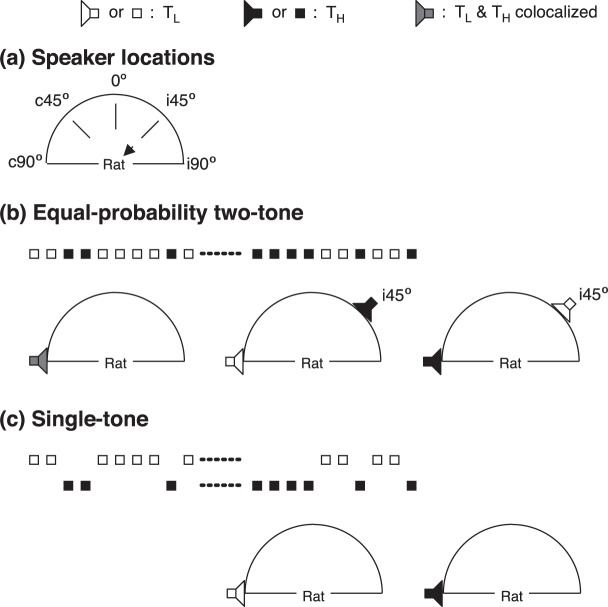
Speaker locations and sound sequences. (**a)** Locations where speakers were calibrated. “” indicates that neural responses were recorded from the right IC. **(b)** Diagrams of an example equal probability two-tone sequence (upper panel) and spatial relationships between two sounds of the sequence (three lower panels). The spatial relationships shown in the figure include two tone bursts colocalized at c90° (left) and separated with one at c90° while the other one at i45° (middle and right). Other relationships, including one at c90° while the other one at c45°, 0°, or i90°, are not shown. **(c)** Diagrams of two single-tone sequences and the location where the sequences were presented (c90°). The two sequences were created by omitting T_H_ (upper sequence) and T_L_ (lower sequence) from the two-tone sequence shown in the upper panel of **(b)**. Speakers and tone bursts associated with T_L_ are indicated by “” and “□”. Speakers and tone bursts associated with T_H_ are indicated by “” and “■”. A speaker used to present both T_L_ and T_H_ at c90° is indicated by “”.

Responses of a neuron to two tone bursts (named as T_L_ and T_H_ elsewhere) of an equal-probability two-tone sequence (Fig. 1b top panel) were obtained when the two sounds were colocalized at c90° (Fig. 1b bottom left panel). They were also obtained when one sound (either T_L_ or T_H_) was at c90° (named as location-fixed sound) while the other one was moved to a non-c90° azimuth (named as location-unfixed sound, see Fig. 1b bottom middle and right panels for the second sound at i45°). No matter whether two sounds were colocalized at c90° or separated by an angle, the response elicited by T_L_ at c90° was not different from that elicited by T_H_ at c90° (Supplementary Fig. S3, two-tailed related-sample Wilcoxon test). When the two sounds were separated by an angle, the response elicited by T_L_ at a non-c90° azimuth was not different from that elicited by T_H_ at the same azimuth (Supplementary Fig. S3, two-tailed related-sample Wilcoxon test). Thus, in the rest of the article when effects of a spatial separation are analyzed in the entire group of neurons, data obtained with T_L_ at a specific azimuth (either c90° or non-c90° azimuth) and data obtained with T_H_ at the same azimuth are combined.

The response of a neuron to a sound at c90° was obtained when the other sound of a two-tone sequence was omitted (Fig. 1c). The response elicited by T_L_ as the remaining sound was not different from the response elicited by T_H_ as the remaining sound (Supplementary Fig. S3, two-tailed related-sample Wilcoxon test). Thus, the effect of omission of a sound on the response to the remaining sound at c90° is analyzed in the entire group of neurons when data obtained with T_L_ omitted and those obtained with T_H_ omitted are combined.

### Effect of spatial separation on the response to a location-fixed sound at c90°

**Figure 2:**
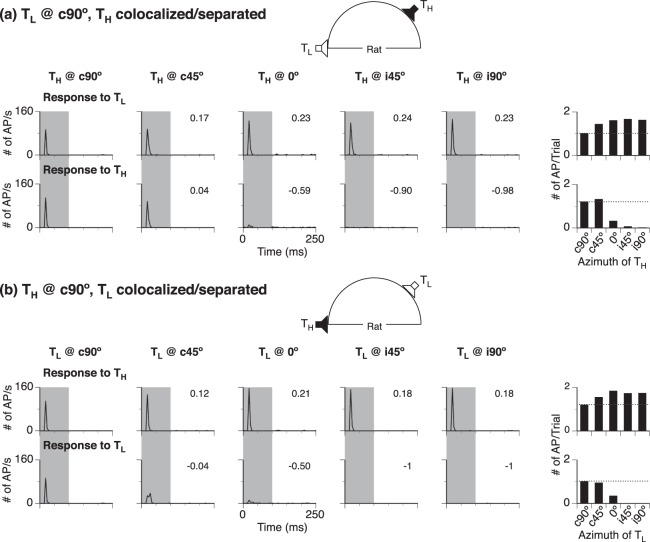
Responses of a representative neuron to two sounds of a two-tone sequence with various spatial relationships between the sounds. (**a)** Results obtained when T_L_ was a location-fixed (c90°) sound while T_H_ was a location-unfixed sound. **(b)** Results obtained when T_H_ was a location-fixed sound while T_L_ was a location-unfixed sound. In both (**a**,**b**), each of the 5 left columns shows two line-time histograms for responses to a location-fixed (top) and a location-unfixed (bottom) sound at a specific angle of separation (azimuth of the location-unfixed sound indicated above the top histogram). Separation-dependent changes of responses to location-fixed and location-unfixed sounds as evaluated by NdR_c90°,Sep_ and NdR_Azimuth_ are indicated in the corresponding upper and lower panels. A shaded area in each column indicates the duration of location-fixed and unfixed sounds of a two-tone sequence. In both **(a**,**b)**, the right column has two bar charts that summarize overall strengths of responses to a location-fixed sound (top) and location-unfixed sound (bottom) at various angles of separation. A horizontal dotted line in a bar chart indicates the height of the first bar. Insets in **(a**,**b)** show speaker arrangements with the location-unfixed sound at i45°. The CF of the neuron: 30.0 kHz.

In many neurons, the overall strength of the response to a location-fixed (c90°) sound was changed when a location-unfixed sound was moved from c90° to another azimuth. For the example shown in Fig. 2, no matter whether the location-fixed sound was T_L_ or T_H,_ the response to the sound was enhanced when a location-unfixed sound was at any non-c90° azimuth (Fig. 2a,b upper panels). The separation-dependent change in the strength of response to a location-fixed sound was evaluated using a normalized-difference-in-response index, NdR_c90°,Sep_. For the neuron in Fig. 2, the index was positive at all angles of separation, reflecting an enhancement in the strength of response. The index was larger when a location-unfixed sound was at 0°, i45°, or i90° than at c45°. Despite an increase in the strength of firing, the transient pattern of firing was not changed by a spatial separation between sounds.

**Figure 3:**
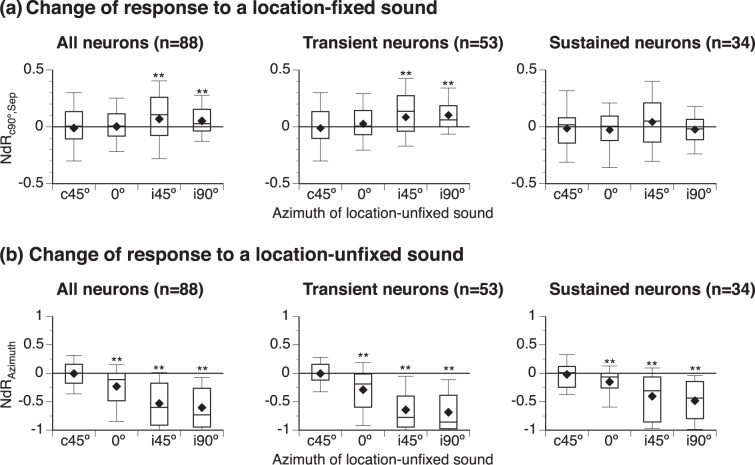
Distributions of NdR_c90°,Sep_ (**a**) and NdR_Azimuth_ (**b**) values. NdR_c90°,Sep_ and NdR_Azimuth_ are indices evaluating changes of responses to a location-fixed and a location-unfixed sound caused by a spatial separation. In both **(a**,**b)**, three charts from left to right show results obtained from the entire population of neurons (n = 88), neurons with transient firing (n = 53), and neurons with sustained firing (n = 34), respectively. Data from a neuron with offset firing are not included in the middle or right charts. At each angle of separation, data obtained with T_L_ presented at c90° while T_H_ at a non-c90° azimuth and with T_H_ presented at c90° while T_L_ at a non-c90° azimuth are combined in a single box. The top and bottom lines of each box indicate the top and bottom quartile while the middle line indicates the median. The two whiskers indicate 90th and 10th percentile, respectively. “♦” indicates the mean. Results of one-sample Wilcoxon signed-rank test for **(a)** left panel: Location-unfixed sound at i45°: Median = 0.102, N = 130, Z = 3.387, p = 0.001; i90°: Median = 0.026, N = 174, Z = 3.693, p < 0.001. Results for **(a)** middle panel: Location-unfixed sound at i45°: Median = 0.136, N = 76, Z = 3.429, p = 0.001; i90°: Median = 0.061, N = 106, Z = 5.410, p < 0.001. Results of one-sample Wilcoxon signed-rank test for **(b)** left panel: Location-unfixed sound at 0°: Median = −0.114, N = 171, Z = −7.073, p < 0.001; i45°: Median = −0.614, N = 130, Z = −9.269, p < 0.001; i90°: Median = −0.736, N = 174, Z = −11.280, p < 0.001. Results for **(b)** middle panel: Location-unfixed sound at 0°: Median = −0.189, N = 105, Z = −5.988, p < 0.001; i45°: Median = −0.775, N = 76, Z = −7.426, p < 0.001; i90°: Median = −0.859, N = 106, Z = −8.863, p < 0.001). Results for **(b)** right panel: Location-unfixed sound at 0°: Median = −0.066, N = 66, Z = −3.846, p < 0.001; i45°: Median = −0.308, N = 54, Z = −5.261, p < 0.001; i90°: Median = −0.437, N = 68, Z = −6.966, p < 0.001. “**” indicates the level of significance at p < 0.005.

At each angle of separation between location-fixed and unfixed sounds, many neurons markedly changed the overall strength of response to a location-fixed sound (Fig. 3a left panel). More neurons displayed positive than negative NdR_c90°,Sep_ indices when a location-unfixed sound was at i45° or i90°, while similar numbers of neurons displayed positive and negative indices when a location-unfixed sound was at c45° or 0° (see Fig. 3 caption for One-sample Wilcoxon signed-rank test results). The distribution of indices was different across angles (Kruskal-Wallis test, χ^2^(3, N = 176) = 14.08, p = 0.003). Further analyses revealed that there were more neurons with transient firing showing increased than decreased responses to a location-fixed sound when a location-unfixed sound was at i45° or i90° (Fig. 3a middle panel, see caption for statistical results). Such a difference wasn’t found in neurons with sustained firing (Fig. 3a right panel). Kruskal-Wallis tests revealed that the distribution of NdR_c90°,Sep_ indices was different across angles of separation in neurons with transient (χ^2^(3, N = 106) = 16.93, p < 0.001) but not sustained (χ^2^(3, N = 68) = 3.23, p = 0.358) firing. Moreover, the distribution was different between transient and sustained-firing neurons (χ^2^(7, N = 174) = 32.55, p < 0.001), with the difference being significant when a location-unfixed sound was at i90° (Conover-Iman *post-hoc* analysis with Bonferroni correction, p < 0.001).

No alterations of the temporal pattern of firing (including subtype) were found in neurons recorded in the present study in response to a location-fixed sound upon spatial separation of the location-unfixed sound. Only minor increases/decreases of the duration of firing were found in neurons with transient or offset firing.

### Effect of spatial separation on the response to a location-unfixed sound

The overall strength of the response to a location-unfixed sound of a two-tone sequence was dependent on the azimuth of the sound. For the neuron shown in Fig. 2, the response was greatly reduced or completely suppressed when the sound was at 0° or an ipsilateral azimuth no matter whether the sound was T_L_ or T_H_ (Fig. 2a,b four lower panels). Such a change in the strength of response was evaluated using an NdR_Azimuth_ index. The index was negative when a location-unfixed sound was at 0° or an ipsilateral azimuth, reflecting a suppression of response. The index was close to -1 when the sound was at an ipsilateral azimuth. The response consistently displayed a transient pattern when it was elicited.

Most of the neurons recorded in the present study displayed a decrease in the overall strength of response to a location-unfixed sound when the sound was moved to 0°, i45°, or i90°. The median NdR_Azimuth_ was significantly smaller than 0 at these azimuths (Fig. 3b left panel, see caption for statistical results). The distribution of NdR_Azimuth_ was different across angles of separation (Kruskal-Wallis test, χ^2^(3, N = 176) = 197.50, p < 0.001). A separation-dependent decrease of the response to a location-unfixed sound was found in both groups of neurons with transient and sustained firing (Fig. 3b middle and right panels, see caption for statistical results). Kruskal-Wallis tests revealed that the reduction was different across angles of separation in both neurons with transient (χ^2^(3, N = 106) = 144.99, p < 0.001) and sustained (χ^2^(3, N = 68) = 58.47, p < 0.001) firing. A difference existed between the two groups (χ^2^(7, N = 174) = 219.32, p < 0.001), with the difference being significant when a location-unfixed sound was at i45° and i90° (Conover-Iman *post-hoc* analysis with Bonferroni correction, p < 0.001 at both azimuths).

All the 54 neurons with transient or offset firing did no alter the pattern (including subtype) of firing in response to the location-unfixed sound when the sound was moved from c90° to another azimuth. Some of these neurons shortened the duration of firing, which accompanied a reduction in the strength of firing, upon a location change of the sound. Thirteen of the 34 sustained-firing neurons altered the pattern of firing in response to a location-unfixed sound when the sound was moved from c90° to another azimuth. Alterations included from one sustained subtype to another subtype (n = 7) and from a sustained type to a transient type (n = 6). The remaining 21 sustained-firing neurons did not show direction-dependent changes in the pattern (including subtype) of firing.

### Changes of responses to location-fixed and location-unfixed sounds compared

**Figure 4:**
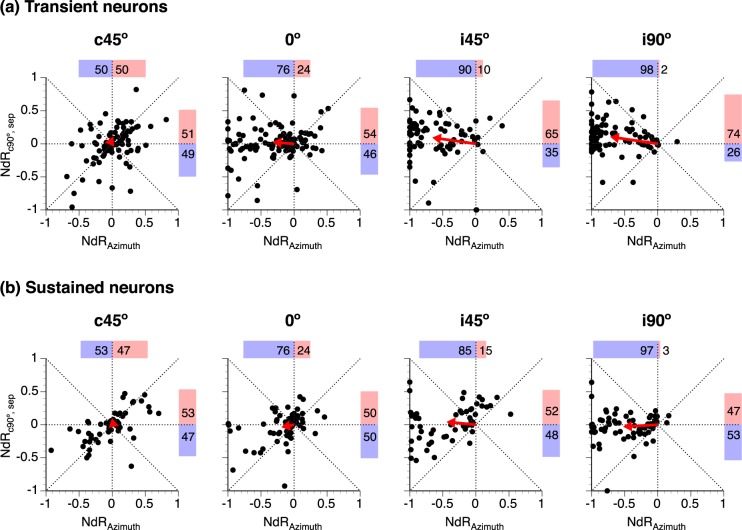
Comparisons between NdR_c90°,Sep_ and NdR_Azimuth_ values in individual neurons. NdR_c90°,Sep_ and NdR_Azimuth_ are indices evaluating changes of responses to a location-fixed and a location-unfixed sound caused by a spatial separation. **(a**,**b)** are results from neurons with transient and sustained firing, respectively. In both **(a**,**b)**, the four panels from left to right show results obtained when the location-unfixed sound was at c45°, 0°, i45°, and i90° (as indicated above each panel). For each group of neurons, data obtained at a single angle of separation with T_L_ at c90° while T_H_ at a non-c90° azimuth and with T_H_ at c90° while T_L_ at a non-c90° azimuth are combined in a single NdR_c90°,Sep_ - R_Azimuth_ plot. A “●” represents a pair of NdR_c90°,Sep_ and NdR_Azimuth_ values from one individual neuron. Horizontal and vertical dotted lines indicate NdR_c90°,Sep_ and R_Azimuth_ values at 0, respectively. “” indicates the mean of the Cartesian vectors of all the data points. Percentages of data points with positive and negative NdR_Azimuth_ values are shown above a dot plot ( and  along with numbers). Percentages of data points with positive and negative NdR_c90°,Sep_ values are shown on the right side of a dot plot ( and  along with numbers).

Separation-dependent changes of responses to location-fixed and unfixed sounds were compared in an NdR_c90°,Sep_ - NdR_Azimuth_ plot at each angle of separation within two groups of neurons with transient and sustained-firing, respectively (Fig. 4). Within each group, moving a location-unfixed sound to c45° caused similar changes of responses to location-fixed and unfixed sounds in many neurons but different changes in other neurons (Fig. 4a,b left panels). Similar numbers of neurons displayed increased and decreased responses to a sound, no matter whether the sound was location-fixed or unfixed. Cartesian vectors were obtained for each data point in an NdR_c90°,Sep_ - NdR_Azimuth_ plot. The mean vector was minuscule for each group of neurons (Fig. 4a,b left panels).

When the location-unfixed sound was at 0°, i45°, or i90°, the distribution of data points in an NdR_c90°,Sep_ - NdR_Azimuth_ plot shifted leftward in both groups of neurons, reflecting a decrease of response to the sound (Fig. 4a,b panels 2–4). For most of the transient-firing neurons, a decrease of response to a location-unfixed sound was accompanied by an increase of the response to a location-fixed sound (Fig. 4a panels 2–4). Thus, the mean Cartesian vector increased its magnitude and reduced its angle. In contrast, similar numbers of sustained-firing neurons increased and decreased responses to a location-fixed sound (Fig. 4b panels 2–4). Thus, the angle of the mean vector remained around 180°. A χ^2^ test indicated that the distribution of data points over the four quadrants of the Cartesian plane was different between transient- and sustained-firing neurons when the relocated sound was at i45° (χ^2^(3, N = 126) = 16.09, p = 0.001) and i90° (χ^2^(3, N = 168) = 20.71, p < 0.001).

### Effect of omission of a sound on the response to a remaining sound

**Figure 5:**
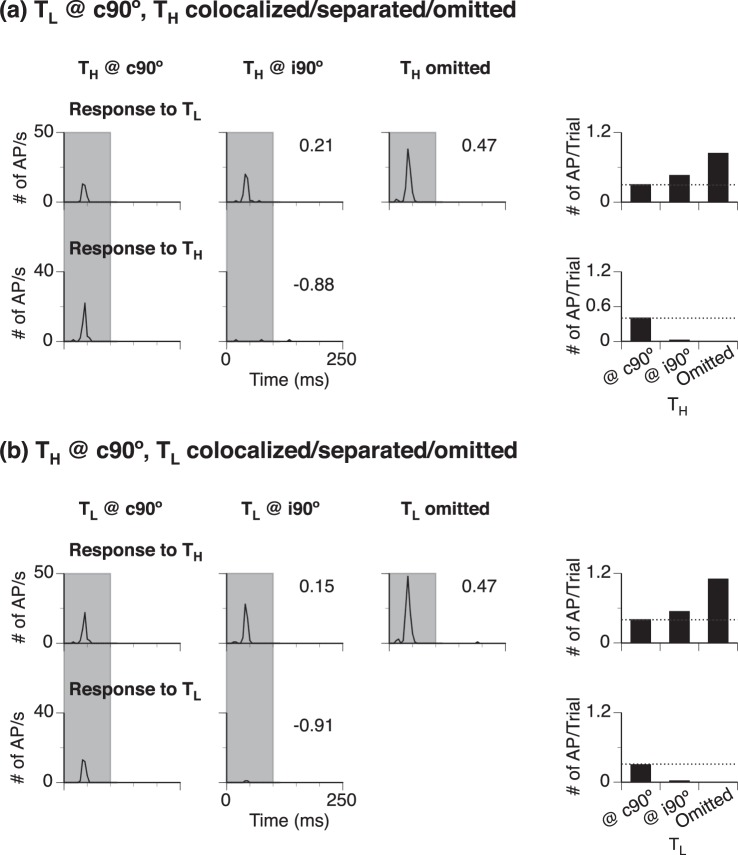
Responses of a representative neuron to a sound at c90° in the presence and absence of the other sound. **(a**,**b)** are results obtained when T_L_ and T_H_ was a location-fixed sound at c90°, respectively. In both **(a**,**b)**, the left and middle columns show line-time histograms for responses to a location-fixed (top) and a location-unfixed (bottom) sound when the second sound was at c90° and i90° (as shown above the column), respectively. Numbers in the top and bottom panels of the second column are NdR_c90°,Sep_ and NdR_Azimuth_ values obtained with the location-unfixed sound at i90°. These numbers evaluate changes of responses to a location-fixed and a location-unfixed sound caused by moving the second sound from c90° to i90°. The line-time histogram in the third column is the response to a location-fixed sound obtained with the other sound omitted (as shown above the column). The change of the response to the location-fixed sound caused by the omission (top first and third panels compared) is evaluated by an NdR_c90°,Omit_ index (shown at the upper-right corner of the third panel). A shaded area in each of the first two columns indicates the duration of location-fixed and unfixed sounds of a two-tone sequence. The right column has two bar charts that summarize overall strengths of responses to a location-fixed sound (top panel) and the other sound (bottom panel). A horizontal dotted line in a bar chart indicates the height of the first bar. The CF of the neuron: 6.0 kHz.

To understand how the response to a sound at c90° was affected by a colocalized second sound, the response to the first sound was obtained when the second sound was omitted (Fig. 1c). The overall strengths of the responses to the first sound in the absence and presence of the second sound were compared and the difference was evaluated using an NdR_c90°,Omit_ index. For the neuron shown in Fig. 5, the response to a c90° sound was enhanced when a colocalized second sound was omitted (Fig. 5a,b right vs. left top histograms). This enhancement was larger than that caused by moving the second sound from c90° to i90° (Fig. 5a,b middle vs. right top histograms). This difference was supported by a comparison between NdR_c90°,Omit_ and NdR_c90°,Sep_ and contrasted with the fact that the second sound did not elicit any firing under both conditions when the sound was omitted and when it was presented at i90°.

**Figure 6:**
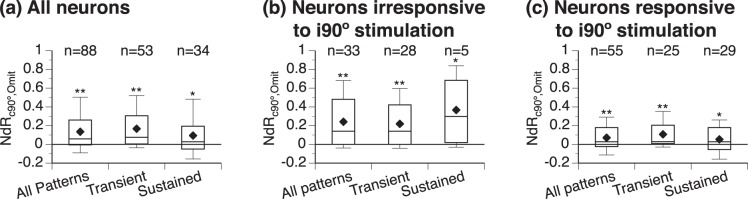
Group result showing NdR_c90°,Omit_ values. NdR_c90°,Omit_ is an index evaluating a change of the response to a sound at c90° caused by omission of a colocalized second sound. **(a**–**c)** show results obtained from the entire group of 88 neurons, 33 neurons that were irresponsive to i90° stimulation, and 55 neurons that were responsive to i90° stimulation. Results were further analyzed in neurons with transient and sustained firing (second and third boxes in **(a**–**c)**). Results from a neuron with offset firing are not included in this further analysis (see numbers in **(a**,**c)**). The number of neurons in each group/subgroup is indicated above a corresponding box. Each box combines data obtained when T_L_ was omitted and T_H_ was omitted. The top and bottom lines of a box indicate the top and bottom quartile while the middle line indicates the median. The two whiskers indicate 90th and 10th percentile, respectively. “♦” indicates the mean. Results of one-sample Wilcoxon signed-rank test for (**a**) first category (all patterns): Median = 0.059, N = 176, Z = 6.619, p < 0.001; second category (transient): Median = 0.076, N = 110, Z = 6.780, p < 0.001; third category (sustained): Median = 0.028, N = 64, Z = 2.451, p = 0.014. Results of one-sample Wilcoxon signed-rank test for **(b)** first category: Median = 0.141, N = 66, Z = 5.500, p < 0.001; second category: Median = 0.141, N = 56, Z = 4.963, p < 0.001; third category: Median = 0.298, N = 10, Z = 2.293, p = 0.022. Results of one-sample Wilcoxon signed-rank test for **(c)** first category: Median = 0.028, N = 110, Z = 3.903, p < 0.001; second category: Median = 0.029, N = 50, Z = 4.446, p < 0.001; third category: Median = 0.028, N = 58, Z = 2.046, p = 0.041 “*” and “**” indicate levels of significance at p < 0.05 and p < 0.001, respectively.

The response to a sound at c90° was enhanced by omission of the other sound in most of the recorded neurons (Fig. 6a). The median NdR_c90°,Omit_ was significantly larger than 0 in the entire group of neurons as well as neurons with transient and sustained firing (One-sample Wilcoxon signed-rank test, see Fig. 6 caption).

Further analyses of the effect of omission of a sound were conducted in two populations of neurons that were irresponsive (n = 33) and responsive (n = 55) to a sound (either T_L_ or T_H_) at i90°, respectively (Fig. 6b,c). Based on the level of spontaneous firing of neurons recorded in the present study, firing below 10 spikes over 100 sound presentations was considered not sound-driven. The two populations of neurons were not different from each other in CF (Mann-Whitney test, U = 841.50, p = 0.511). Regardless of pattern of firing, the overwhelming majority of the neurons that were irresponsive to i90° stimulation increased responses to a sound at c90° when the other sound was omitted (Fig. 6b, see caption for One-sample Wilcoxon signed-rank test results). Of the neurons that were responsive to i90° stimulation, the majority increased responses to a sound at c90° when the other sound was omitted (Fig. 6c first category, see caption for One-sample Wilcoxon signed-rank test results). The percentage of neurons showing increases was larger in transient- than sustained-firing neurons (Fig. 6c second vs. third category). Between neurons that were irresponsive and responsive to i90° stimulation, an omission-dependent enhancement was larger in the first population (Fig. 6b,c first category, Mann-Whitney test, U = 2501.00, p < 0.001). This difference between the two populations was primarily due to neurons with sustained (Fig. 6b,c third category, U = 156.00, p = 0.02) instead of transient firing (Fig. 6b,c second category, U = 1113.00, p = 0.069).

**Figure 7:**
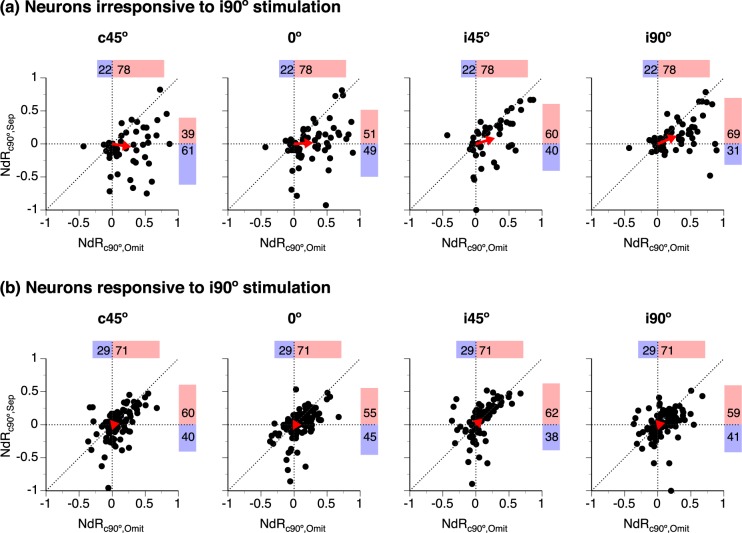
Comparisons between NdR_c90°,Sep_ and NdR_c90°,Omit_ values in individual neurons. NdR_c90°,Sep_ and NdR_c90°,Omit_ are indices evaluating changes of the response to a location-fixed (c90°) sound caused by moving a colocalized second sound to another azimuth and omission of the second sound, respectively. (**a**,**b)** are results from neurons that were irresponsive and responsive to an i90° sound, respectively. For both **(a**,**b)**, the same set of NdR_c90°,Omit_ values is used in all the four panels. NdR_c90°,Sep_ values in each panel were obtained when the location-unfixed sound was at a specific azimuth (as indicated above the panel). Results obtained with T_L_ at c90° and those obtained with T_H_ at c90° are combined in one NdR_c90°,Sep_ - NdR_c90°,Omit_ plot. Each “●” represents a pair of NdR_c90°,Sep_ and NdR_c90°,Omit_ values from one individual neuron. Horizontal and vertical dashed lines indicate NdR_c90°,Sep_ and NdR_c90°,Omit_ values at 0, respectively. The 45° diagonal line indicates equal values of NdR_c90°,Sep_ and NdR_c90°,Omit_. In each panel, “”indicates the mean of the Cartesian vectors of all the data points. Percentages of data points with positive and negative NdR_c90°,Omit_ values are shown above a dot plot ( and  along with numbers). Percentages of data points with positive and negative NdR_c90°,Sep_ values are shown on the right side of a dot plot ( and  along with numbers). Results of related-sample Wilcoxon signed-rank test for **(a)** when a location-unfixed sound was at c45°: N = 54, Z = −5.191, p < 0.001; at 0°: N = 65, Z = −5.096, p < 0.001; at i45°: N = 46, Z = −3.381, p = 0.001; at i90°: N = 66, Z = −3.696, p < 0.001.

An NdR_c90°,Sep_ - NdR_c90°,Omit_ plot is used to compare changes of the response to a location-fixed (c90°) sound caused by separating a colocalized second sound and omitting the second sound (Fig. 7). For neurons that were irresponsive to i90° stimulation, most data points are on the right side of the y-axis, supporting that the response to a c90° sound was enhanced by omission of the other sound in most neurons (Fig. 7a all panels). For the same population of neurons, there are more data points above the x-axis at larger angles of separation (Fig. 7a). The distribution of data points (above vs. below the x-axis) is different across angles of separation (Kruskal-Wallis test with Conover-Iman *post hoc* analysis with Bonferroni correction, χ^2^(3, N = 66) = 14.22, p = 0.003). The angle of the mean Cartesian vector was increased at larger angles of separation. The enhancement of the response to a c90° sound caused by omission of the other sound was larger than that caused by moving the second sound to another azimuth (including i90°) in most neurons (first quadrant in all panels of Fig. 7a, see caption for results from related-sample Wilcoxon signed-rank tests). This difference in enhancement deserves special attention, as no response was elicited by the second sound under both conditions.

**Figure 8:**
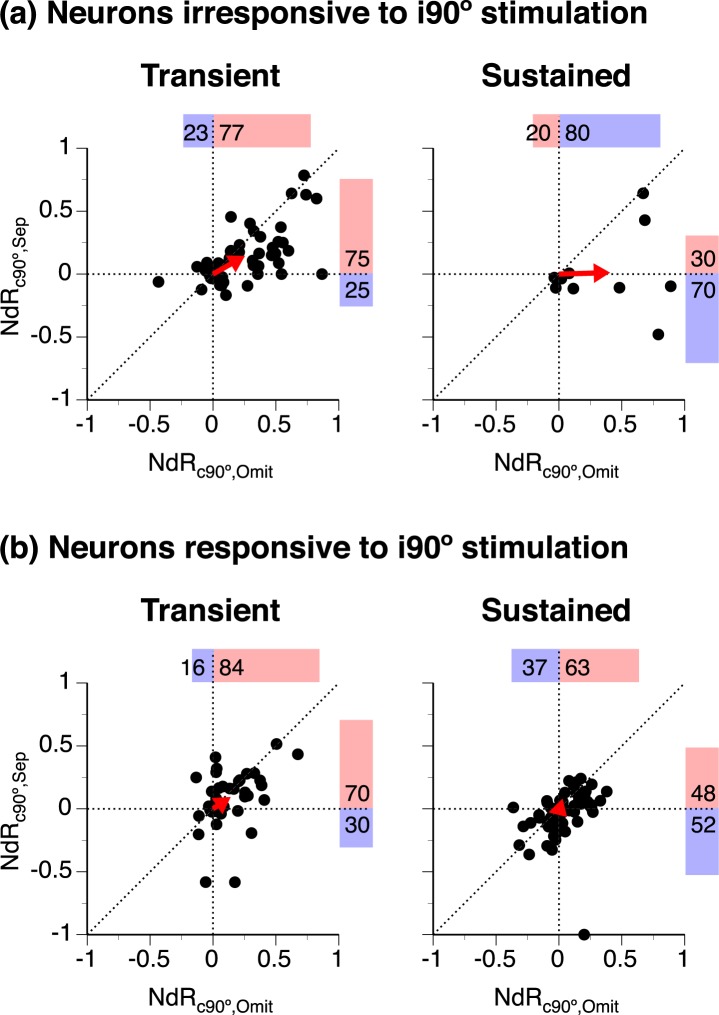
Comparisons between NdR_c90°,Sep_ and NdR_c90°,Omit_ values in individual neurons with transient and sustained firing. NdR_c90°,Sep_ and NdR_c90°,Omit_ are indices evaluating changes of the response to a location-fixed (c90°) sound caused by moving a colocalized second sound to i90° and omission of the second sound, respectively. **(a**,**b)** are results from neurons that were irresponsive and responsive to an i90° sound, respectively. Within each of the two groups, results are presented separately for neurons with transient (left panel) and sustained (right panel) firing. Each “●” represents a pair of NdR_c90°,Sep_ and NdR_c90°,Omit_ values from one individual neuron. NdR_c90°,Sep_ values were obtained with the location-unfixed sound at i90°. Results obtained with T_L_ at c90° and T_H_ at c90° are combined in one NdR_c90°,Sep_ - NdR_c90°,Omit_ plot. Horizontal and vertical dashed lines in each panel indicate NdR_c90°,Sep_ and NdR_c90°,Omit_ values at 0, respectively. The 45° diagonal indicates equal values of NdR_c90°,Sep_ and NdR_c90°,Omit_. In each panel, “” indicates the mean of the Cartesian vectors of all the data points. Percentages of data points with positive and negative NdR_c90°,Omit_ values are shown above a dot plot ( and  along with numbers). Percentages of data points with positive and negative NdR_c90°,Sep_ values are shown on the right side of a dot plot ( and  along with numbers).

Neurons irresponsive to i90° stimulation included those with transient and sustained firing. There were more transient-firing neurons increased than decrease responses to a c90° sound when the other sound was separated (see Fig. 8a left panel for the second sound at i90°). This difference was not apparent in sustained-firing neurons.

Of those responsive to i90° stimulation, more neurons increased than decreased the response to a c90° sound when the other sound was omitted (Fig. 7b all panels). Within the same group, more neurons increased than decreased responses to a c90° sound when the other sound was moved from c90° to another azimuth (Fig. 7b panels 1–4). However, the distribution of NdR_c90°,Sep_ values was not significantly different across angles of separation (Kruskal-Wallis test with Conover-Iman *post hoc* analysis with Bonferroni correction, χ^2^(3, N = 110) = 6.898, p = 0.075).

In each panel of Fig. 7b, most of the data points are distributed along or close to the 45° diagonal, with many of the points clustered around the origin of the Cartesian plane. Cartesian vectors of data points in each panel of Fig. 7b had smaller magnitudes than those in a corresponding panel of Fig. 7a (Kolmogorov-Smirnov test, first column: D = 0.282, N = 54,96, p = 0.008; second column: D = 0.277, N = 65,108, p = 0.004; third column: D = 0.309, N = 46,86, p = 0.006; forth column: D = 0.300, N = 66,110, p = 0.001). Results suggest responses to a c90° sound were similarly affected by omission and separation of the other sound and the effects were generally mild in most neurons that were responsive to i90° stimulation.

Of neurons that were responsive to i90° stimulation, those with sustained firing had data points distributed along the 45° diagonal in an NdR_c90°,Sep_ - NdR_c90°,Omit_ plot and close to the origin of the Cartesian plane (see Fig. 8b right panel for located-unfixed sound at i90°). It was primarily these neurons that caused relatively clustered distribution of data points in Fig. 7b. Data points from transient-firing neurons were more widely distributed (see Fig. 8b left panel for relocated sound at i90°).

## Discussion

The present study revealed that responses of an IC neuron to two tone bursts of an equal-probability two-tone sequence were dependent on the spatial relationship between the sounds. For many neurons, when one of the two sounds that were colocalized at the contralateral ear (c90°) was moved to an ipsilateral azimuth the response to the sound with a location change was reduced while the response to the sound with a fixed location was enhanced (Figs 2–4). The enhancement was particularly large in neurons with transient firing and not responsive to i90° stimulation (Figs 7 and 8). Thus, the detection of a recurring target sound by IC neurons was affected by a colocalized recurring interfering sound; and spatial separation between the two sounds enhanced the detection of the target sound.

Increase of the response to a location-fixed (c90°) sound upon spatial separation of the other sound is a major finding of the present study. Similar effect of separation was observed in a few previous studies conducted in the rat’s and cat’s auditory cortex^34,35^. In these studies, responses of a neuron were elicited by two repetitively presented independent Gaussian noise bursts with one at a fixed azimuth while the other one either colocalized or separated. Presentations of the two sounds formed a sequence with an alternating pattern. For many neurons, the response to a location-fixed sound was increased upon separation of the other sound, especially when the response to the separated sound was reduced. Such a separation-dependent increase of the response to a location-fixed sound was not apparent when the same noise-burst sequence was used to elicit responses in the rat’s IC^35^. Thus, a difference in the effect of spatial separation existed between the previous^35^ and the present study in the rat’s IC. It is unknown whether such a difference was due to disparities between acoustic stimuli used in the two studies. These disparities include the quality of sound (two different tone bursts vs. two independent Gaussian noises), regularities of sounds (random vs. alternating) in a sequence, and specific location of sounds (colocalized at c90° vs. other angles).

The increase of the response to a location-fixed (c90°) sound upon spatial separation of the other sound suggested that the response to the first sound was masked when the second sound was colocalized and released from masking when the second sound was spatially separated^2,36^. Neural substrates of spatial release from masking have been studied in the cat’s and frog’s IC^27,28^. In the cat, neurons that selectively responded to low-frequency sounds displayed an improved population masked threshold when a target sound was spatially separated from a continuous noise masker^28^. In the frog, many neurons in the IC (especially those with transient firing) displayed improved signal detection threshold when a target sound was separated from a concurrent noise masker^27^. These separation-dependent neurophysiological changes are different from that observed in the present study. Thus, neural substrates underlying spatial release from masking are possibly species specific. Alternatively, they may be dependent on acoustic characteristics of target/masking sounds including frequency (low vs. high), temporal relationship (simultaneous vs. non-simultaneous), and number of incidences (recurring vs. single-event).

The reduction of the response to a sound that was moved from c90° to an ipsilateral azimuth (i.e., location-unfixed sound) reflected the spatial tuning characteristic of an IC neuron. This directional dependent change was consistent with previous results from the rat’s IC showing that responses of neurons to a dichotic stimulus decreased when the interaural-level difference of the stimulus became favoring the ipsilateral ear^20,21,23,30,37^. It was also consistent with findings from other species showing that high frequency-sensitive IC neurons typically have spatial receptive fields located in the contralateral acoustic hemifield^24,25,38–40^. In the present study, the direction-dependent change of the response to a location-unfixed sound was observed in the existence of a location-fixed sound. Such a change might be different from that evaluated without the second sound^33,41–44^. Further research is needed to determine how the special tuning characteristic evaluated by a recurring sound is influenced by another recurring sound.

Neural mechanisms underlying effects of spatial separation between two tone bursts on responses to the sounds likely involve excitatory/inhibitory binaural interaction. Major excitatory inputs to the rat’s IC are from the contralateral cochlear nucleus^15^. Inhibitory inputs are from the ipsilateral superior olivary complex and ventral nucleus of the lateral lemniscus as well as both ipsi- and contralateral dorsal nucleus of the lateral lemniscus^16–19,45^. Inhibitory inputs can also be from within the IC^46,47^. As a result, most neurons in the rat’s IC are excited by stimulation of the contralateral ear and inhibited by simultaneous stimulation of the ipsilateral ear^20,21,23,30,37^.

Reduction of the response to a sound that was moved from c90° to an ipsilateral azimuth could be due to weakening of excitatory inputs and/or strengthening of inhibitory inputs driven by the sound. The reduction of response was greater in neurons with transient than sustained firing (Fig. 3b), which agreed with a previous result showing that IC neurons with regular firing typically had weak sensitivities to the interaural-level difference^48^. Dissimilarities exist across IC neurons with different firing patterns in the degree and time course of binaural inhibition^22,23,48^. These dissimilarities might have caused the differences in the dependence of response on sound direction (under free-field stimulation) and interaural-level difference (under closed-field dichotic stimulation).

Factors causing enhancement of the response to a location-fixed (c90°) sound upon spatial separation of the other sound might have included a decrease in the level of adaptation. When both tone bursts were at c90°, a quick succession of excitatory inputs driven by a two-tone sequence might have led to strong adaptation and weak responses to both sounds following the first few stimuli within the sequence^49–53^. Moving one sound to an ipsilateral azimuth could have reduced excitatory inputs driven by the sound, which might have lowered the level of adaptation and enhanced the response of the neuron to the sound that remained at c90°.

Other factors causing separation-dependent enhancement of the response to a location-fixed sound might have included alteration in the inhibitory effect generated by neurons in the superior paraolivary nucleus. Neurons in this structure receive excitatory inputs driven by the contralateral ear and provide GABAergic outputs to the ipsilateral IC^54,55^. Many of these neurons generate offset firing in response to a tone burst^56,57^. Thus, an inhibitory aftereffect can be generated by a leading contralateral sound through the superior paraolivary nucleus to inhibit the response of an IC neuron to a trailing contralateral sound. This inhibitory aftereffect likely plays a key role in generating forward masking^58–60^. Over a two-tone sequence with both sounds (T_L_ and T_H_) presented at c90°, each of the 200 stimuli could have produced an inhibitory effect on an IC neuron through the superior paraolivary nucleus, which could have decreased mean responses elicited by both sounds. Moving one sound to an ipsilateral azimuth could have diminished the inhibitory inputs generated by the sound and consequently enhanced the response to the sound that remained at c90°.

The enhancement of the response to a fixed-location sound at c90° caused by moving the other sound from c90° to i90° was typically not as large as that caused by omission of the second sound (Figs 7 and 8). This difference existed even in the subgroup of neurons that were irresponsive to i90° stimulation (Figs 7a and 8a). This fact suggested that the response to a sound at c90° could have been inhibited by another sound at i90°.

Our results suggest that moving a location-unfixed sound from c90° to i90° likely generated multiple effects including increase in ipsilateral inhibition, reduction in adaptation, and reduction in superior-olivary-nucleus-related contralateral inhibition. These effects interacted with each other in shaping the response to a sound at c90°. When the first effect exceeded the total of other two effects, the response could be reduced rather than enhanced (Figs 7 and 8 data points below the x-axis).

In some neurons, especially those with sustained firing and responsive to i90° stimulation, separation- and omission-dependent changes of the response to a location-fixed sound were similar to each other (Fig. 8b right panel). For these neurons, ipsilateral inhibition generated by a location-unfixed sound did not seem to play a major role in shaping the response to the fixed-location sound.

It should be noted that the inhibitory effect generated by a location-unfixed sound could influence the response to a location-fixed sound only if the effect lasted longer than the interval between two consecutive stimuli (150 ms in this study). Long lasting ipsilateral inhibition in the IC has been revealed by previous *in vivo* neurophysiological studies^30,61–65^ and supported by *in vitro* neurophysiological recordings^66^. Such inhibition can suppress the response to a contralateral sound that is presented tens or even hundreds of milliseconds later^30^.

Our results along with those from the frog’s IC^27^ indicate that neurons with transient firing are particularly suitable for detecting spatial separation between sounds. Among the special characteristics that transient-firing neurons have, brief firing may enable these neurons to better utilize the onset interaural-level difference of a sound and avoid binaural adaptation^67^. Further investigation should be conducted to find whether transient-firing IC neurons can facilitate spatial release from masking at a behavioral level. Such investigation is of particular significance as onset interaural-level difference is important for hearing in an environment with multiple asynchronous sounds^68,69^. This difference is reset every time when a new sound is generated.

As a summary, the present study has revealed that the spatial relationship between two qualitatively different and temporally asynchronous recurring sounds affects the responses of midbrain auditory neurons to the sounds. The effect of spatial separation is likely related to changes in excitatory/inhibitory interaction in these neurons. Our results are important for understanding neural mechanisms responsible for spatial hearing.

## Materials and Methods

### Animal preparation

Experiments were conducted using adult male Wistar albino rats (*Rattus norvegicus*, 250–600 g) obtained from Charles River Canada Inc. (St. Constant, QC). Surgical anaesthesia was induced by ketamine hydrochloride (60 mg/kg, i.m.) and xylazine hydrochloride (10 mg/kg, i.m.) and maintained by supplementary injections of ketamine hydrochloride (20 mg/kg, i.m.) and xylazine hydrochloride (3.3 mg/kg, i.m.).

A craniotomy was made on the right side of the skull for placing a recording electrode into the IC. The skull was cemented onto a head bar attached to a custom-made holding device. A recording electrode was held by a custom-made clamp attached to the slave cylinder of a Model 650 micropositioner, which was fitted onto a micromanipulator of a Model 900 stereotaxic instrument (Kopf Instruments, Tujunga, CA). Instruments were positioned in such a way that acoustic shadows and reflections were minimized. The rat was placed in a Model CL-15A LP acoustic chamber (Eckel Industries, Morrisburg, ON). Experimental protocols were approved by the University of Windsor Animal Care Committee in accordance with the guidelines of the Canadian Council on Animal Care. All experiments were performed in accordance with the relevant guidelines and regulations.

### Acoustic stimulation

Sound waveforms were generated using a System 3 real-time signal processing system controlled by a personal computer running OpenEx software (Tucker-Davis Technologies, Alachua, FL). Sounds were presented using two Model FF1 free-field speakers (Tucker-Davis Technologies, Alachua, FL). Each speaker was held by a custom-made mounting device and could be positioned at any azimuthal location 50 cm away from the midpoint of the interaural line. Each speaker was calibrated over 100 and 65,000 Hz at five azimuths (Fig. 1a) using a model 4135 microphone and a model 2608 measuring amplifier (Brüel & Kjaer, Dorval, QC). These azimuths included the midline of the frontal field (denoted by 0°) and 90° and 45° on the contra- and ipsilateral side of the recording site (denoted by c90°, c45°, i45°, and i90°).

### Recording electrode and procedures

Action potential discharges were recorded extracellularly from single neurons in the right IC using single-barrel glass micropipettes filled with 3 M NaCl (tip diameter ~1.5 μm, impedance 5~10 MΩ). The electrode was located within the coronal plane at a 30° angle relative to the midsagittal plane. It was 4.0 mm lateral and 0.4 mm rostral in reference to lambda. While electrophysiological activities were monitored audio-visually, Gaussian noise bursts at 60 dB SPL were presented from a loudspeaker at c90° to search for an auditory neuron. Neural signals were amplified by a 2400 A preamplifier (Dagan, Minneapolis, MN) and sampled at 24.4 kHz using the System 3 real-time signal processing system.

Upon isolation of a single auditory neuron, the characteristic frequency (CF, the frequency at which the neuron displayed the lowest threshold) and threshold at CF were determined using tone bursts presented at c90°. A threshold was the lowest sound-pressure level at which a tone burst with 5-ms rise/fall phases and a 90-ms plateau presented at 4/s elicited action potential discharges over at least 3 of 10 presentations of the tone burst.

Neurons recorded in the present study displayed transient, sustained, or offset patterns of firing in response to a CF tone burst presented at c90° (Supplementary Fig. S2). Transient patterns included onset and fast-adapting subtypes. An onset subtype had action potential discharges only over a period shorter than 20 ms at the onset of a tone burst (Supplementary Fig. S2). A fast-adapting subtype had discharges that gradually attenuated from a high level to the level of spontaneous firing before the offset of a tone burst. An offset pattern had discharges at the offset of a tone burst. Only if a neuron generated transient or offset firing over the range from the threshold at CF to the highest level tested (typically 85 dB SPL) was the neuron classified into the transient or the offset category. Sustained patterns included primary-like, pauser, and build-up/later subtypes. A primary-like subtype had strong transient firing at the onset of a tone burst followed by reduced firing over the rest of the sound without an interruption, while a pauser subtype had early strong firing and late reduced firing separated by a brief pause. A build-up/late subtype had firing that was initiated after a long delay and ended at the offset of the sound. Neurons that were classified into the sustained category included those that consistently generated sustained firing over the range from the threshold at CF to the highest level tested (typically 85 dB SPL). They also included those that generated sustained firing at some intensities but transient firing at other intensities.

An equal probability two-tone sequence (Fig. 1b upper panel) was created for the isolated neuron. Such a sequence contained 200 stimuli, each of which was one of two tone bursts named as T_L_ and T_H_, respectively. The frequency of T_L_ (named as *f*_*L*_) was lower than CF, while the frequency of T_H_ (named as *f*_*H*_) was higher than CF. The center frequency of *f*_*L*_ and *f*_*H*_ (i.e., (*f*_*L*_ × *f*_*H*_)^1/2^) was at the CF and the difference between the two frequencies (i.e., (*f*_*H*_ − *f*_*L*_)/(*f*_*H*_ × *f*_*L*_)^1/2^) was at 0.10. Each tone burst had 5 ms rise/fall phases and a 90 ms plateau. T_L_ and T_H_ was at the same sound-pressure level (typically at 10–30 dB above the threshold at CF at c90°) at which each sound at c90° could elicit a suprathreshold but not saturated response. For neurons that displayed sustained firing at some levels but transient firing at other levels, the level was chosen to ensure that a sustained response was elicited by each sound when it was presented at c90°. Within a two-tone sequence, the two tone bursts were presented in a random order with each sound presented at a 50% probability. The 200 stimuli were presented at a constant rate of 4/s.

Responses to a two-tone sequence were first recorded when T_L_ and T_H_ were presented from a single loudspeaker at c90° (colocalized, Fig. 1b left-bottom panel). Responses to the sequence were then recorded when one tone burst was presented from the loudspeaker at c90° (named as location-fixed sound) while the other one was presented from another loudspeaker located at c45°, 0°, i45°, or i90° (named as location-unfixed sound). Responses to the two sounds that were separated were then compared with the responses to the same sounds that were colocalized to study how a spatial separation between two sounds affected the response to the sounds. At each angle of separation there were two sound arrangements, i.e., T_L_ at c90° while T_H_ at a non-c90° azimuth and T_H_ at c90° while T_L_ at a non-c90° azimuth (See Fig. 1b middle- and right-bottom panels for a location-unfixed sound at i45°).

A single-tone sequence was created by omitting one tone burst from a two-tone sequence and was presented at c90° (Fig. 1c) to elicit a response. This response to the remaining tone burst was compared with the response to the same sound in a two-tone sequence (with two sounds colocalized at c90°). The dependence of the response to a sound on a colocalized sound was then determined.

### Data analysis

The strength and temporal pattern of firing of a neuron in response to a tone burst in a two-tone or single-tone sequence were analyzed using spikes elicited by 100 presentations of the sound. For all the neurons within transient and sustained responses, spikes elicited by each sound presentation were counted over a 120 ms period starting from the onset of the presentation. These neurons did not generate sound-driven firing beyond this period. For the neuron with an offset response, spikes were counted over a 50 ms period starting from the offset of a sound presentation. The mean number of action potentials elicited by 100 presentations of a tone burst was used to represent the overall strength of the response to the sound. Action potentials elicited by 100 presentations of a tone burst were used to create a peri-stimulus time histogram (see Supplementary Fig. S2). A firing rate was calculated for each of the 5-ms time bins using the number of spikes elicited within the bin. Firing rates obtained from all bins over the entire cycle of sound presentation/data acquisition (250 ms) were used to create a line-time histogram to indicate the temporal change of the strength of firing (e.g., Figs 2 and 5).

The overall strengths of the responses elicited by two tone bursts that were spatially separated were compared with those of responses evoked by the same tone bursts that were colocalized to evaluate the effect of a spatial separation. An NdR_c90°,Sep_ index was used to evaluate the effect on the response to a c90° sound:

$$Nd{R}_{c{90}^{^\circ },Sep}=\frac{{R}_{c{90}^{^\circ }}(\alpha )-{R}_{c{90}^{^\circ }}(c90^\circ )}{{R}_{c{90}^{^\circ }}(\alpha )+{R}_{c{90}^{^\circ }}(c90^\circ )}$$

where R_c90°_(*α*) and R_c90°_(c90°) are the strengths of responses elicited by a location-fixed (c90°) tone burst when the location-unfixed tone burst was at azimuth *α* (two sounds separated) and c90° (two sounds colocalized), respectively. At each angle of separation, two NdR_c90°,Sep_ values were obtained for each neuron for responses to T_L_ and T_H_ as a location-fixed sound at c90°, respectively.

The effect of a spatial separation on the strength of response to the location-unfixed tone burst of a two-tone sequence was evaluated using an NdR_Azimuth_ value:

$$Nd{R}_{Azimuth}=\frac{{R}_{Azimuth}(\alpha )-{R}_{Azimuth}(c90^\circ )}{{R}_{Azimuth}(\alpha )+{R}_{Azimuth}(c90^\circ )}$$

where R_Azimuth_ (*α*) and R_Azimuth_ (c90°) are the strengths of response to a location-unfixed sound when it was at azimuth *α* and c90°, respectively. At each angle of separation, two NdR_Azimuth_ values were obtained for each neuron for responses to T_L_ and T_H_ as a location-unfixed sound, respectively.

The response evoked by a tone burst in a single-tone sequence presented at c90° was compared with the response evoked by the same sound in a two-tone sequence with two sounds colocalized at c90°. An NdR_c90°,Omit_ value was calculated to evaluate how the response to a tone burst was affected by a colocalized second tone burst.:

$$Nd{R}_{c{90}^{^\circ },Omit}=\frac{{R}_{1}-{R}_{2}}{{R}_{1}+{R}_{2}}$$

where R_1_ and R_2_ are the strengths of responses to a tone burst when it was presented in a single tone sequence and a two-tone sequence, respectively.

Non-parametric statistical tests were used in the analysis as normal distributions of data were not found. The one-sample Wilcoxon signed-rank test was used to determine if the median of a dataset was different from 0. The two-tailed related-samples Wilcoxon test was used to compare two measurements within individual neurons. The Mann-Whitney U test was used to compare measurements from two different groups of neurons. The Kolmogorov-Smirnov test was used to compare measurements from two different groups of neurons when the shape of distribution of measurements was different between the groups. The Chi-Square test was used to compare distributions of data points over the Cartesian plane. The Kruskal-Wallis test was used to compare multiple groups of data, and the Conover-Iman *post-hoc* analysis was used to identify a difference between any two of these groups. Bonferroni correction was used to correct for type I error. Statistical analysis was conducted using the SPSS 17.0 software (IBM Corporation, Armonk, NY).

## Supplementary information

Supplementary figures

## Data Availability

The datasets generated and analysed during the current study are available from the corresponding author on reasonable request.
