# Supplementary material for: Responses of neurons in the rat’s inferior colliculus to a sound are affected by another sound in a space-dependent manner
Source: Sci Rep. 2019 Sep 26;9:13938. doi: 10.1038/s41598-019-50297-8 (PMC6763450; doi:10.1038/s41598-019-50297-8)
Supplement: Supplementary file 1 — Supplementary figures [file 41598_2019_50297_MOESM1_ESM.pdf]

Responses of neurons in the rat's inferior colliculus to a sound are affected by another sound in a space-dependent manner

Mathiang G. Chot, Sarah Tran, Huiming Zhang*

Department of Biomedical Sciences, University of Windsor, Windsor, Ontario, Canada N9B 3P4

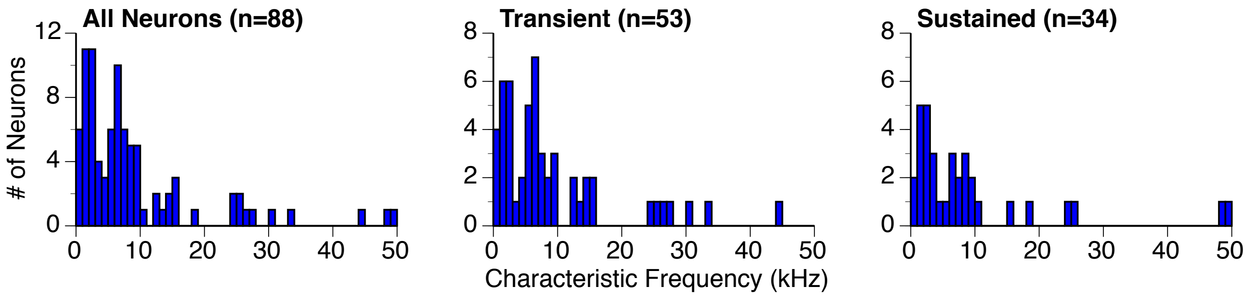

Supplementary Figure S1. Distribution of characteristic frequencies within the entire group of 88 neurons recorded in the present study (left panel), as well as 53 neurons with transient firing (middle panel) and 34 neurons with sustained firing (right panel). The left panel includes a neuron with offset firing. This neuron is not included in either the middle or the right panel. The characteristic frequency of a neuron was determined using tone bursts presented from a loudspeaker located at the ear contralateral to the neuron (c90°).

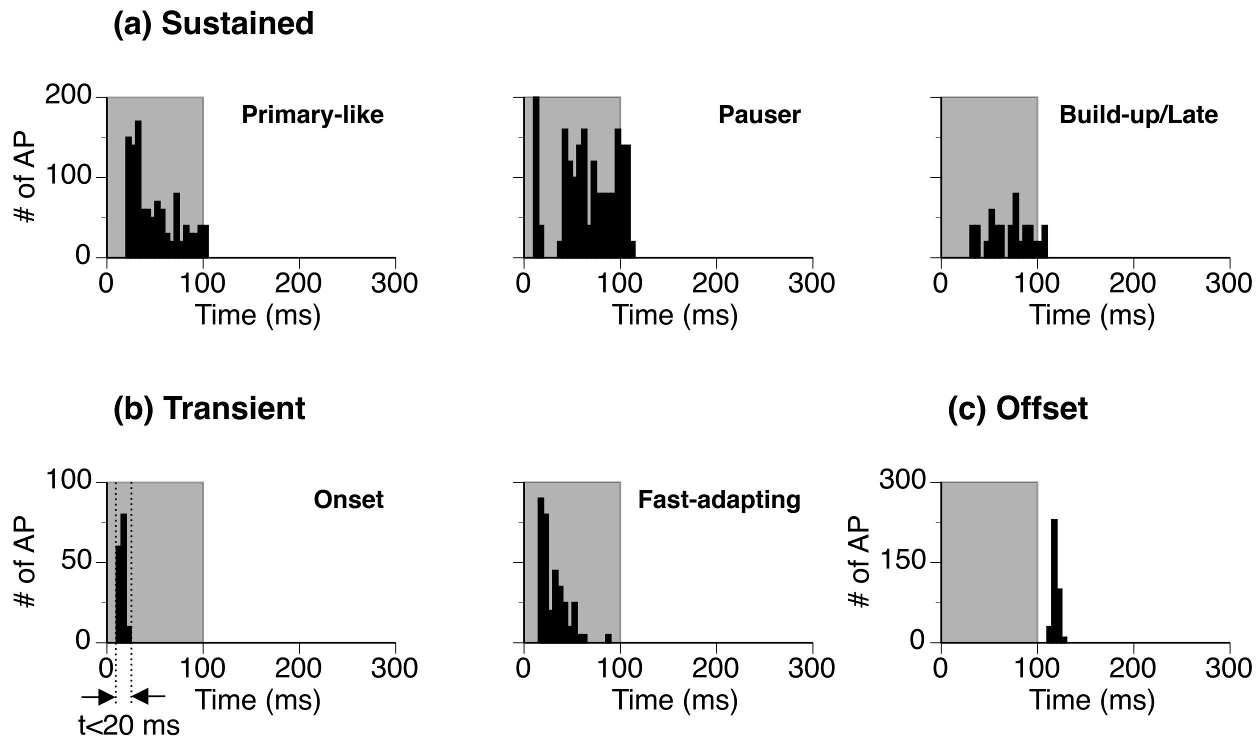

Supplementary Figure S2. Temporal patterns of firing generated by 6 representative neurons recorded in the present study. Major patterns included sustained **(a)**, transient **(b)**, and offset **(c)** types. The sustained type included primary-like (**(a)** left panel), pauser (**(a)** middle panel), and build-up/late (**(a)** right panel) subtypes, while the transient type included onset (**(b)** left panel) and fast-adapting (**(b)** right panel) subtypes. In the **(b)** left panel, two vertical dotted lines along with two horizontal arrowheads indicate the duration of firing (<20 ms). A shaded area in each panel indicates the duration of a sound presentation. Bin widths: 5 ms.

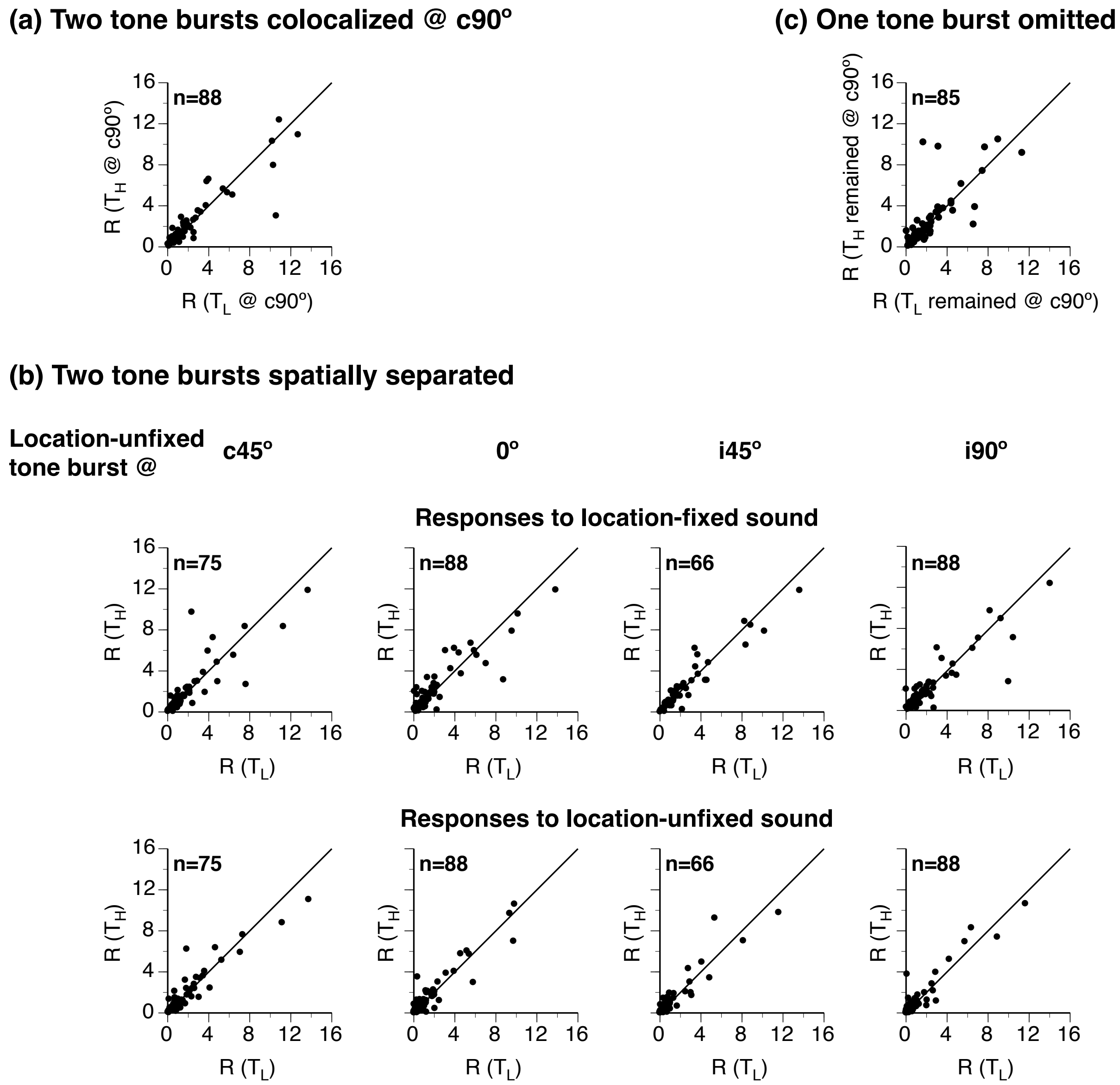

Supplementary Figure S3. Group results comparing responses evoked by T_L and T_H presented at the same azimuth. Results were obtained when one sound (either T_L or T_H) of a two-tone sequence was presented at a fixed location at c90°, while the other sound was either colocalized at c90° **(a)** or separated from the first sound **(b)** or omitted **(c)**. Each column in **(b)** shows results obtained at a specific angle of separation, with the location of the location-unfixed sound indicated above the panel. The upper panel of each column compares responses to T_L and

T_H at c90°, while the lower panel compares responses to T_L and T_H at a non-c90° azimuth. "R" in the title of an axis represents the strength of response (i.e., mean number of action potentials per sound presentation). In each plot, a "●" represents results from one individual neuron. A diagonal line indicates equal strengths of responses to T_L and T_H. The number of neurons from which results were obtained is indicated in the top-left corner of each plot.
